# Regulatory mechanisms to create healthier environments: planning appeals and hot food takeaways in England

**DOI:** 10.1177/17579139231187492

**Published:** 2023-08-12

**Authors:** CL O’Malley, AA Lake, HJ Moore, N Gray, C Bradford, C Petrokofsky, A Papadaki, S Spence, S Lloyd, M Chang, TG Townshend

**Affiliations:** Centre for Public Health Research, School of Health & Life Sciences, Teesside University, Middlesbrough TS1 3BA, UK; Fuse, The Centre for Translational Research in Public Health, Newcastle upon Tyne, UK Email: c.o’malley@tees.ac.uk; Centre for Public Health Research, School of Health & Life Sciences, Teesside University, Middlesbrough, UK; Fuse, the Centre for Translational Research in Public Health, Newcastle upon Tyne, UK; Fuse, the Centre for Translational Research in Public Health, Newcastle upon Tyne, UK; School of Social Sciences, Humanities & Law, Teesside University, Middlesbrough, UK; School of Social Sciences, Humanities & Law, Teesside University, Middlesbrough, UK; Centre for Public Health Research, School of Health & Life Sciences, Teesside University, Middlesbrough, UK; Fuse, the Centre for Translational Research in Public Health, Newcastle upon Tyne, UK; UK Health Security Agency, London, UK; Centre for Exercise, Nutrition and Health Sciences, School for Policy Studies, University of Bristol, Bristol, UK; Fuse, the Centre for Translational Research in Public Health, Newcastle upon Tyne, UK; Human Nutrition Research Centre, Population Health Sciences Institute, Faculty of Medical Sciences, Newcastle University, Newcastle upon Tyne, UK; Fuse, the Centre for Translational Research in Public Health, Newcastle upon Tyne, UK; Public Health South Tees, Middlesbrough, UK; Department of Health and Social Care, Office for Health Improvement and Disparities, London, UK; Fuse, the Centre for Translational Research in Public Health, Newcastle upon Tyne, UK; School of Architecture, Planning & Landscape, Newcastle University, Newcastle upon Tyne, UK

**Keywords:** public health policy, public health, obesity, health policy, environment

## Abstract

**Aims:**

To explore existing regulatory mechanisms to restrict hot food takeaway (HFT) outlets through further understanding processes at local and national levels.

**Methods:**

The Planning Appeals Portal was utilised to identify recent HFT appeal cases across England between December 2016 and March 2020. Eight case study sites were identified using a purposive sampling technique and interviews carried out with 12 professionals involved in planning and health to explore perceptions of and including factors that may impact on the HFT appeal process. Additionally, documents applicable to each case were analysed and a survey completed by seven Local Authority (LA) health professionals. To confirm findings, interpretation meetings were conducted with participants and a wider group of planning and public health professionals, including a representative from the Planning Inspectorate.

**Results:**

Eight case study sites were identified, and 12 interviews conducted. Participants perceived that LAs would be better able to work on HFT appeal cases if professionals had a good understanding of the planning process/the application of local planning policy and supplementary planning documents; adequate time and capacity to deal with appeals cases; access to accurate, robust, and up to date information; support and commitment from elected members and senior management; good lines of communication with local groups/communities interested in the appeal; information and resources that are accessible and easy to interpret across professional groups.

**Conclusions:**

Communication across professional groups appeared to be a key factor in successfully defending decisions. Understanding the impact of takeaway outlets on health and communities in the long term was also important. To create a more robust appeals case and facilitate responsiveness, professionals involved in an appeal should know where to locate current records and statistical data. The enthusiasm of staff and support from senior management/elected officials will play a significant role in driving these agendas forward.

## Introduction

Obesity is a significant health and social problem. Addressing factors that contribute to high energy intakes and subsequently excess weight gain is an important public health challenge. ‘Dramatic actions’ are needed, globally, to address food environments and thereby impact on the rise in obesity, cardiovascular disease and type 2 diabetes.^1^ A population approach (as opposed to individual level approaches) is to address the environments that promote less healthy eating and high energy intake.

There is an urgent need to shift focus to a more upstream (or macro-level) whole systems approach to obesity^2^, using cross-sector and multi-agency working to consider the multiple factors that influence individual determinants. Examples of upstream approaches could be through use of planning^3^ or taxation of less healthy foods.^4^ This research focuses on the Planning Appeals process in England, which is managed by The National Planning Inspectorate (PINS).^5^ The environment has been acknowledged as a determinant of health,^6^ and that (1) eating out of home is positively associated with risks of overweight and obesity^7^ and (2) that food eaten out of home is usually less healthy and provides a higher energy contribution from fat compared to food eaten at home.^8^ This potential role of the built environment and planning in creating healthier communities was reflected in the 2012 National Planning Policy Framework (NPPF) for England,^9^ which sets out planning policies and how they are expected to be applied. The NPPF and associated Planning Practice Guidance was revised in 2019 and now includes more detail about promoting health and wellbeing, for example by citing access to healthier food, green space and building environments that promote walking and cycling as specific aims.^10^

We can define the food environment as any opportunity to obtain food; it includes physical, socio-cultural, economic and policy influences at both micro and macro levels.^11^ The broader food environment includes the home food environment, food policies and school food policies in addition to the neighbourhood food environment.^12^ This research focuses on the neighbourhood food environment and specifically hot-food takeaways, within the broader context of obesogenic environments. Takeaway and fast food, a ﬁxture of our diet, is usually nutrient poor and energy dense.^8,13^ There is a ‘concentration effect’, with a clustering of these fast-food outlets and neighbourhood exposure being greater in more deprived areas.^14,15^

Policy documents have highlighted the role that Local Authorities (LAs) have in tackling obesity.^16–18^ An umbrella literature review^19^ assessed the impact of the built and natural environment on health. The review concentrated on five key built environment topics: neighbourhood design, housing, healthier food, natural and sustainable environment, and transport. These are environmental issues that can be shaped by planners and have the potential to influence health.

There has been a recent interest in the role of LAs in shaping the food environment,^20^ particularly via engaging small businesses^21^ and planning departments,^22^ but also the wider neighbourhood food environment. The latter is defined as a mixture of retail outlets (e.g. small convenience stores and supermarkets) as well as restaurants and take-away (‘fast food’) outlets and is *not* limited to the residential neighbourhood.^11^ The neighbourhood food environment influences individual food choice and food intake through the concept of food *access*. Access, in terms of the food environment, includes five dimensions which are: *availability, accessibility, affordability, acceptability*, and *accommodation*.^23^ Planning legislation can influence both availability and accessibility of these outlets.

Using The Town and Country Planning (Use Class) Order 1987, outlets are classified according to the use class order of the premises they occupy, dependent upon their primary operating model and premise size (Classifications of interest are in Box 1), and in 2005, a specific ‘A5’ Hot Food Takeaways was introduced. From September 2020, the classification of Hot Food Takeaways (HFTs) was changed to the sui generis class (meaning ‘in a class of its own’).

**Box 1:** The town and country planning (use class) order^a^.

A1, retail – includes sandwich bars and internet cafes A3, restaurants and cafes A5, hot food takeaways

a From September 2020, A1 and A3 have been replaced by Class E, and A5 has changed to Sui Generis.

An increasing number of LAs are using Supplementary Planning Documents (SPDs) to control fast food outlet proliferation.^24^ Barking and Dagenham was the first LA to introduce an SPD in 2010 which gave weight to health impacts, focusing on both public health and nutrition.^22^ Such an approach is now suggested by Public Health England (PHE, now Office for Health Improvement and Disparities (OHID)) for LAs to influence the out-of-home food environment,^19^ alongside use of the local plans, joint strategic needs assessments, joint health and wellbeing strategies, sustainability and transformation plans and the use of Health in all Policies.

**Figure 1:**
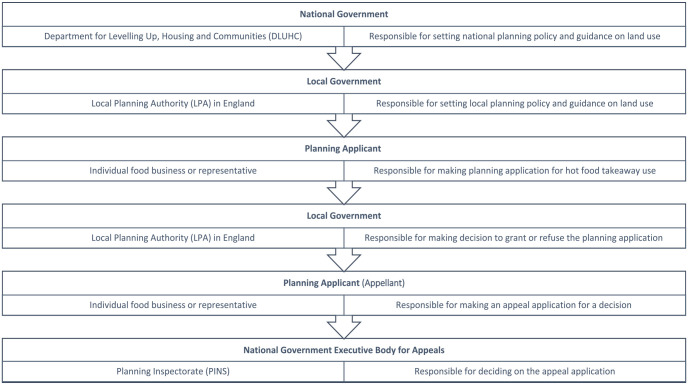
End to end process for decision-making on hot food takeaways

Work published in 2019 by Keeble et al.^25^ showed that 51% of LAs in England have a planning policy to restrict HFTs, and 34% of these (56 LAs) state protecting public health as a key driver. However, the effectiveness of these policies will ultimately depend on their successful implementation. In part, successful implementation will depend on enforcement when a prospective HFT owner (the ‘appellant’) appeals against an initially rejected planning application. Final decisions are then taken out of local hands and are made instead by PINS (the focus of this work) based on representations by the LA and the appellant. Decisions that are upheld are those that are in agreement with the initial LA decision, while decisions that are dismissed are those that overturn the initial decision. The end to end process for decision-making on HFTs is outlined in Figure 1. This research aims to build on previous evidence which explores decisions made by PINS^26^ including perspectives from a variety of professionals involved in HFT planning appeals, providing a more holistic insight into the process.

## Method

**Figure 2:**
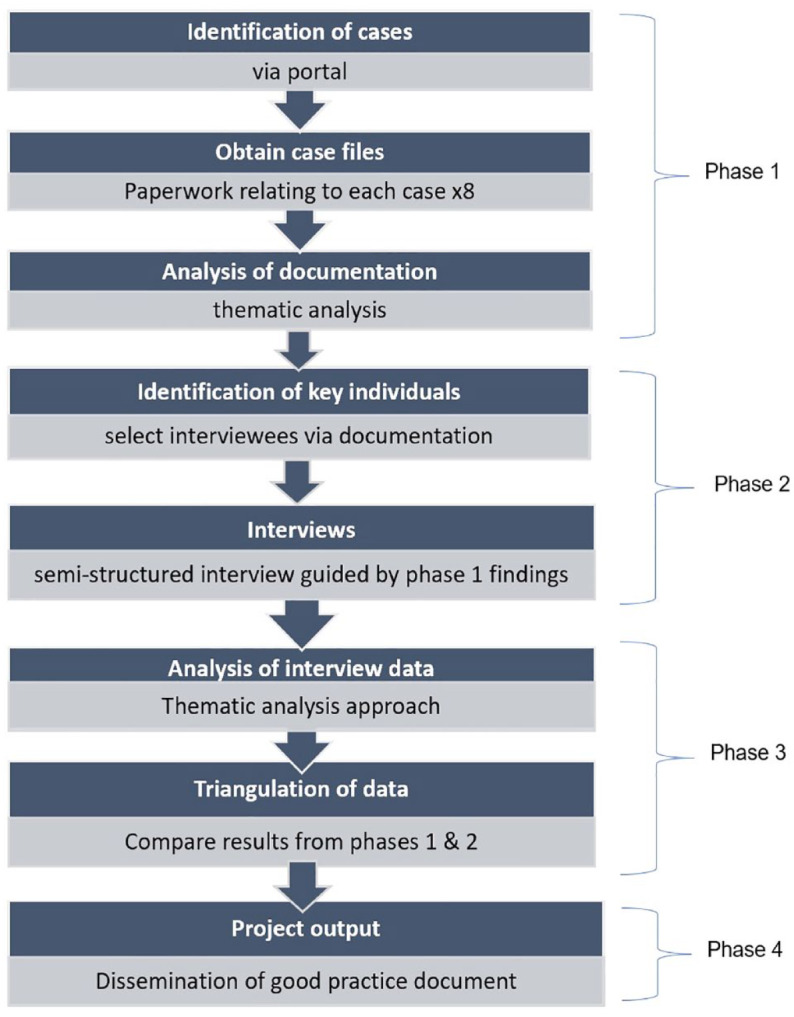
Project flow diagram

See Figure 2 for the project flow diagram. The Planning Appeals Portal (PAP) [https://appealfinder.co.uk/] was utilised to identify recent case studies across England. 47 HFT appeal cases across 34 LAs were found, spanning from 2016 to 2020. From these, eight case study sites were identified to further explore information considered in HFT appeal cases. The typology of action was applied as developed by Keeble et al.^25^ to select studies. Cases were selected using a purposive sampling technique which is particularly useful in obtaining information that contributes to a deeper understanding of the topic of interest.^27^ Cases were selected that; mentioned HFT appeals and health, cited health and/or obesity as a factor in the case decision and had textual information in relation to health and/or obesity as an addition to the final appeal decision. This included documents such as planning documents, policies, residential and/or business letters and LA responses etc. An even number of both cases that were upheld and dismissed were selected, ensuring each region was represented including North East and Yorkshire, South West, Midlands and London.

Data collection for each site took part in three phases:

### Phase 1: identification of case study sites and documentary analysis

Characteristics of the 47 cases were obtained. Extracted data included the appeal decision (upheld/dismissed), Indices of multiple deprivation (IMD) decile, authority tier, and whether any SPDs and Local Plan policies were cited. Documentation from each case was examined, evidence, where available, was extracted and analysed to provide contextual data for each case study site. For example, this included; appeal type, agent involvement, total number of documents attached to each case, key documents that contained health-related information, priority placed on health, application for costs, whether other cases were cited and use of statistics, reports, maps, academic documents and whether Local Plans and SPDs were in place at the time the case decision was made. Descriptive statistical analysis was conducted to explore possible associations.

### Phases 2 and 3: interviews and online survey

Interviews were carried out with nine planners, one public health professional, one public representative, and a representative from the Planning Inspectorate (independent from the case studies) between October 2020 and January 2021. All interviews were carried out over Microsoft Teams or via telephone using a predetermined semi-structured interview guide, the development of which was directed by key stakeholders involved in the project and using evidence obtained within associated case documents (identified in phase 1). This ensured that questions were relevant to case players, added local context to the format and structure of the interviews and allowed for further exploration of any barriers and facilitators to the appeals decision making, identified in phase 1. Each participant was asked a core set of questions related to the appeals process in general, supplemented with case specific questions, where appropriate. Participants provided written and verbal consent prior to taking part and all interviews were recorded, transcribed and anonymised. Ethical clearance was approved by Teesside University’s School of Health and Life Science Committee (Ref: 150/19).

We were unable to interview any business owners. The interview phase was carried out during the COVID-19 pandemic and availability of participants was significantly impacted by this. This also posed recruitment challenges and an online survey was launched via Jisc Online Surveys to boost responses from across LAs (containing very similar questions to those used in interviews). The questionnaire was sent to those 47 LAs across England via our steering group members (however, professional groups already interviewed were excluded from the mailout).

### Data analysis

Documentary case information for each case study site was analysed using a content analysis technique and interview data thematically analysed.^28,29^ NVivo V.10 was used to assist with the organisation and analysis of data.^30^ Analysis was performed by two researchers (CO and CB) and findings discussed with two other researchers (HM and AL). Data was drawn together from each phase using a triangulation technique and narratively synthesised to identify both barriers and facilitators to the appeals decision-making process and to make specific recommendations to inform the development of a successful appeal case.

## Results

### Phase 1 (identification of appeal cases)

#### Characteristics of the 47 HFT cases

Forty-seven appeal cases (mentioning HFTs) between 2015 and 2020 were identified across England; 21 were upheld (45%) and 26 dismissed (55%). Most cases were based in more deprived areas (IMD deciles 1–4 *n* = 39), although there was no association between deprivation and appeal decision (based on descriptive statistics). Twenty-five cases were decisions made by LAs in the North of England, compared to 22 in the South. However, this figure is not indicative of proportionality of appeal cases but attributed to the sampling approach taken which allowed for a geographical spread of appeal cases. Twenty-seven cases were under a unitary authority system, with 20 having two-tier systems. Cases under a two-tier system appeared marginally more likely to be upheld (55%), compared to unitary authorities (37%). Thirty-nine cases were aided by SPDs.

#### Documentary analysis of case-study sites (*n* = 8)

From these 47 cases (see above), eight were purposefully selected as case study sites. Documents were available to review for six of the eight case study sites in urban/Metropolitan Unitary Authorities in England (two in the North East, two in the West Midlands, two in London and one each in West Yorkshire and the South West). The number of documents attached to each case ranged from 10 to 86. There appeared to be duplicates or missing documents in some cases, however all those available were reviewed for content and data extracted based on the following criteria; location, decision, appeal type, agent (Y/N), total no. of documents, key documents that included health related information, order health placed as a priority, other stated cases (Y/N), statistics used (Y/N), SPD (Y/N), Local Plan (Y/N), academic documents (Y/N), maps (Y/N), details on statistics, details of academic resources, application for costs included (Y/N). Characteristics of the eight case study sites are outlined in Table 1.

**Table 1:** Details of data collection for the eight case study sites

LA	Decision	Year	Sup. planning doc.	Interview			Doc. analysis	Regions
				Planning	Public health	Business		
North East A	Dismissed	2020	Yes	No	Yes	No	Yes	North East & Yorkshire
North East B	Upheld	2019	Yes	Yes	No	No	Yes	
West Yorkshire	Dismissed	2018	Yes	No	No	No	Yes	
West Midlands A	Dismissed	2018	Yes	Yes	No	No	Yes	Midlands
West Midlands B	Upheld	2016	No	Yes	No	No	No	
South West	Upheld	2017	Yes	No	No	No	Yes	South West
London A	Dismissed	2020	No/London Plan	Yes	No	No	No	London
London B	Upheld	2020	No/London Plan	Yes	No	No	Yes	

LA: local authority.

Appeal types were predominantly proposed changes of use, from class A1 to A5 (*n* = 5) (see Box 1). Two of the remaining cases were proposed erection of new units (one involving demolition of an existing site) and the remaining case was a change of use from A3 to A5 (see Box 1). Planning Agents were used in 5/8 cases, of which 3/5 were upheld. Key documents ranged from the decision notices only (in cases where there were no other documents available or very little information pertaining to health) to an array of documents, including officer reports, planning statements, email correspondence, appeal statements, letters of support and decision notices. Health was cited as the primary reason for the decision made in 3/8 cases (one case was dismissed, and two cases were upheld). In all remaining cases (*n* = 5), health was cited as a secondary issue/ reason or cited within ‘other matters’ concerning the cases. Reasons which superseded health issues were; effect on living conditions, the vitality and viability of city centres, character and highway safety.

Health statistics were used to support six of the cases; this included statistics from PHE (now OHID) documents, local obesity trends and statistics, % of HFT’s locally, National Obesity Observatory stats, National Child Measurement Programme (NCMP) data, Office for National Statistics (ONS) data, ward population data and retail survey data. PHE reports (now OHID), LA reports (including council committee reports) and healthy weight strategies, as well as the Foresight report were referred to in four of the cases. The remainder had no documents attached so it was unclear if reports had been used. Only one case included the use of academic publications to support their case (used in a case that was dismissed (North East A). SPDs were present in 5/8 cases. Of these three were dismissed and one upheld.

### Phase 2 (interviews)

The results from the interviews have been divided into themes and subthemes.

#### Perceived role of the planning inspectorate

##### Decision making throughout the process

The Planning Inspectorate (PINS) are the decision-makers in the appeals process. Individual Inspectors are appointed to make decisions on behalf of the Secretary of State (SoS); however, the SoS can step in to recover for determination if deemed appropriate, although this is very rare. When an Inspector makes a decision, it potentially becomes a material consideration in subsequent cases, allowing appellants and planning authorities to use them as a comparison and to argue for consistency in decision-making over similar issues. The appeal process was perceived as confusing and difficult to navigate for some, especially to the public and to those new to the practice, although procedural guidance is published and available to view on the PINS website this was not referred to in any of the interviews:

*It was exhausting . . . I was tearing my hair out. I enjoyed the process, but it was very very hard going*. (ID 9)

##### Only consider evidence presented to them

It was frequently noted how the PINS will only consider evidence if it is presented directly to them and that this was the responsibility of the case specific officer. It was stated that appellants should not assume that PINS know anything about the available evidence, requiring a systematic and thorough approach to pulling together all available evidence to support a case:

*All the information needs to be out there, clearly so that the inspectorate can make the decision with ease*. (ID 8)

Moreover, it was suggested that the appeals process was one that was generally fair and clear from a planning perspective:

*I think the planning inspectorate operate in a very clear and transparent manner, but they only consider evidence that is put before them.* (ID 5)

This was confirmed through speaking with a representative from the Planning Inspectorate who saw themselves a separate entity with the task of providing an impartial decision based on all available evidence:

*Inspectors are there to provide rigor and to review the evidence on an objective basis, not just to say, oh, because it’s agent X or company X, therefore it must be right. They are there to look, think independently for themselves and look to see if there are holes in that, in that evidence.* (ID 10)

#### Relevance and prioritisation of evidence

##### General consensus over certain types of evidence

Across interviews with all three professional groups (public health, planning, and the Planning Inspectorate), there was agreement that certain types of evidence were prioritised over others. Some forms of evidence were perceived to be undisputable and essential to a successful appeal (e.g. reference to Local Policy), while others were seen as ‘anecdotal’, unreliable, and to generally be avoided (e.g. the views of the public).

##### Local plan/ policy and statistical (data) evidence

Two forms of evidence were highly cited by respondents: reference to the local plan and or planning policy (*n* = 5) and statistical evidence or quantitative data (*n* = 6). These forms of evidence were regarded as the ‘gold standard’ and often necessary for an appeal to be successful. For councils based in London, the London Plan was perceived as carrying significant weight in comparison to the local plan which was considered more generic:

*The London Plan. . .there’s a bit more detail about, in particular hot food takeaways and obesity and that’s given more standing.* (ID 5)

The most common example regarding planning policy was the minimum distance new HFTs must be from local schools; usually described as 400 to 800 metres, a distance which is usually deemed to be a 5-to-10-min walk. One participant noted trying to measure the distance between a new potential outlet and the local school but being unable to establish that the distance was below 800 m:

*We said it was 816, we measured differently, but we could never get it under the 800, no matter how we tried.* (ID 9)

Interviewees often referred to statistics as a separate entity to that which cited planning policy; with participants consistently stating that statistical evidence was often fundamental to a planning appeal case, and always preferable if available:

*You’ve always got to have a statistic to back it up.* (ID 3)

Again, a reoccurring example of statistics used was the NCMP, with planning authorities outlining that obesity in a certain area may already be above the national average, with local plans sometimes restricting the opening of new HFTs in such zones. Wider determinants of health could also be drawn out of Joint Strategic Needs Assessment or PHE (now OHID) reports, which were perceived as useful in identifying priority areas of concern.

##### Academic, authoritative and expert evidence

Academic, authoritative or expert evidence was likewise cited as useful in a planning appeal. Examples varied from peer-reviewed academic papers (particularly systematic reviews), data from PHE (now OHID), government publications, legislation, administerial statements, relevant authoritative groups or professionals, even comments from The House of Commons. However, this type of evidence was not referred to as frequently, and when referenced, it was often to endorse or complement a prior argument, which would have already been supported by one of the previous two primary sources of evidence.

*I suppose, sort of systematic reviews, academic papers, PHE sort of data and any kind of supplementing evidence to kind of support that* . . . (ID 2)

##### ‘Anecdotal’ evidence

Anecdotal evidence was rarely mentioned explicitly, however, evidence that did not fit into any of the previous categories was commonly described as ‘anecdotal’ and perceived to be less powerful. All professional groups stated a preference for material, binary evidence with little room for subjectivity, as such evidence was deemed the most difficult to argue against. Anecdotal evidence included the views of local residents:

*. . . facts of the case . . . they’re looking to base a decision on, on um, quantifiable evidence, not on anecdotal hearsay.* (ID 1)

##### Notable discrepancies

The previous forms of evidence discussed were largely agreed upon in terms of the amount of weight that is applied to them within the appeals process. However, there were some differences in opinion between professional groups regarding SPDs and the role of public health evidence. Both the Planners and the Planning Inspectorate were keen to point out that SPDs were often applied incorrectly in practice and were not considered to be particularly strong forms of evidence, while public health professionals tended to perceive SPDs as key to a successful appeal.

Most participants described public health evidence as being a fundamental part of their appeal cases and was cited as underpinning planning policies and constituting the majority of supporting information:

*100% relevant. The public health evidence underpins our planning policy evidence base* … (ID 4)

However, there were some exceptions. Notably some planners stated that HFT appeals are rarely refused solely on a public health basis, but rather for other reasons such as highway safety, noise disturbances, or previous, similar planning decisions:

*Well, in terms of most appeals we had in terms of hot food takeaways, not a great deal because the refusals have been on other grounds as well as health.* (ID 1)

Another notable point was that although the Planning Inspectorate was viewed across all professional groups as fair and neutral; the perception from public health was that they didn’t think the inspectorate gave enough weight to public health evidence, or that they had to go out of their way to ‘state the obvious’, in that a new takeaway would be unhealthy and cause harm.

On the other hand, Planners and the Inspectorate were keen to point out that planning policy and the appeals process is not designed solely with public health in mind, and that achieving public health objectives is not as simple as limiting the number of HFT:

*I think there seems to be an expectation from the public health side of things that planning will provide policy, so that we’ll deliver whatever their aiming to achieve, y’know what I mean, like restricting take-aways will be the end of it from a health point of view, and of course planning is not actually designed to do that.* (ID 1)

#### Perceived factors to compiling a successful HFT appeal case

##### Communication

Communication played a significant role in putting an appeal case together. Cross-department working, knowing who to approach in an LA as well as where to find outside sources of information that could add value to a case (such as academic papers, reports and statistics) were believed to facilitate the process. Absence of working in a multidisciplinary way was perceived to impact on the ability to collate evidence for a case:

*Some officers aren’t so good at, knowing where all the information is, and in some local authorities, the teams don’t work together.* (ID 4)

##### Accessibility of evidence and data

Access to both national and local data was considered important. Health statistics were cited as being central by some. This was also believed to be important even if there were already local plans and policies in place:

*It’s all well and good having the policy but it needs the evidence as well to back it up. So, having access to the public health team and the public health evidence is a really, really relevant part of the appeals process.* (ID 5)

##### Storage and updating of information

Having up-to-date information at hand was stated by some as being useful in helping to collate and respond to cases, making it less time-consuming to collect. It also meant that information was at hand and relevant. The additional effort to prepare and update information periodically was perceived as something that was worthwhile and beneficial in the long term, creating a stronger evidence base to draw upon when needed, in turn strengthening cases:

*So, as a side matter, we always thought, if we keep on top of the hot food takeaway evidence, then when it comes to examination, all we are ever doing is just updating, we’re not starting from scratch . . . each year we have a lot of Excel spreadsheets to plough through, to update this paper but each year we’ll learn something new, or iron out a little crease, that, the more we do it, the more perfect it is. Whereas if we just left it, from 2017, for five years, we’d be like – how do we record this again?* (ID 4)

##### Format of evidence

It was not only important to have this information readily available but it was also important that it was usable. Often it was deemed to be in a format which was tricky to interpret or make sense of, and therefore could be difficult to use:

*Often, I find that when I’ve had to do research for like health matters and planning, the data is there but it’s very hard to kind of interpret or it’s going across multiple sources.* (ID 5)

##### Understanding the importance of health

Understanding the importance of health and the implications of health on the wider planning agenda was considered valuable. Several participants felt this acknowledgement was lacking across professional groups, including planners. There were suggestions for additional training on the topic.

##### Passion, drive and commitment from elected members

The passion and drive of LA staff came through strongly in interviews. Individuals who appeared passionate about the topic were proactive, knew where to find data and had a good knowledge of their local area:

*So, having the ability to talk to other people that also have conversations with other people, helps bring some of that information back to me, and it helps me feel more empowered to drive the policy forward, and not just give in and say ah go on we’ll have another hot food takeaway, because I fully understand that it is having an impact on kids’ lives.* (ID 5)

Some spoke of how elected members often provided input when cases were subject to a hearing. They spoke of decisions being dependent on the evidence presented by the officers and the elected members’ perspective on this:

*The officers make recommendations and the elected members make a decision based on officer advice and their own interpretation of the case. They often don’t go with the officer advice and that’s one of those things.* (ID 6)

### Phase 3 (online survey)

In total, there were seven respondents to the survey, four of whom were from a planning background, and three from public health. Participants were based across various regions including North East and Yorkshire (3), the Midlands (1), London (1), and the South West (2). Two of the participants stated they were involved in HFT cases. Participants perceived that Planning Inspectors tended to have a focus on enabling economic activity, citing a lack of public health consultation and involvement in the process. Others suggested that the process was not in any way unfair, and that losing a case was nothing to do with the fairness of the system, but rather the balance of evidence on offer.

When asked what participants thought constituted evidence within a planning appeal, answers included the number of takeaways already open within the area, particularly around schools, as well as childhood obesity rates, mortality and morbidity data, socio-economic data, and academic evidence relating to behaviour and fast food. Participants also noted that evidence which is directly related to the locality and/or considers the economic aspects of a planning appeal carries the most weight in the appeals process.

Local Planning Policies, SPDs, and general articles which support the impact of hot-food takeaways were all listed as the health-related policies and documents which are frequently referenced in appeal cases. However, views on the relevance of health-related policy in an appeals case was mixed, with three stating ‘sometimes’ or ‘maybe’, and four answering ‘very’ relevant. Ways in which public health agencies could support the appeals process included lobbying for health to be a more material consideration in planning and providing more robust evidence which links the proliferation of takeaways with obesity rates. Suggestions for any changes that could enhance the appeals process ranged from speeding up the process, creating stronger national policies, and the NPPF explicitly stating that health is a material consideration for planning.

## Discussion

To the best of our knowledge, there are no studies that have explored the role of the Planning Inspectorate (at a national level) in planning for health. The aim of this research was to explore existing regulatory mechanisms to restrict HFT outlets through further understanding processes at local and national levels. It also aimed to build on previous research carried out which questioned the importance of heath, the use of policy documents and other evidence within the decision-making process.^26^ Findings from the case studies demonstrated that a takeaway’s potential impact on the local populations’ health was often cited as a reason against the change of land use or the construction of a new takeaway. However, this rationale was frequently referred to as a secondary concern and did not appear to make for a more successful defence. This is in line with the findings from the interviews and online surveys, where public health professionals felt the impact on health was not only based on evidence but common sense, describing a need to ‘state the obvious’ at every appeal. This led to a substantial level of tension with planning professionals on the other hand, who believed that public health professionals did not fully understand the role of planning, which was fundamentally about regulating land use and not about health policy.

These Internal relationships within LAs were important, including the relationships between different departments, how effectively they work together (particularly public health and planning), resources available and the size of the authority in terms of staff numbers. Interest from senior management and particularly political drive from elected councillors can be key in setting a LA’s approach and providing resources. Where there is political commitment, a LA is more likely to find the time and resources to prepare a detailed case. Additionally, a solid legislative base for a LA case is vital, specifically the statutory local plan rather than, for example, supplementary planning documents.

There is minimal information available relating to the planning process in terms of HFT appeal cases and no other studies looking at the National Planning Inspectorates role within this process. However, in 2019 the House of Commons outlined the planning appeals process (in general), key players in the system and routes of access to further understand and challenge decisions.^31^ While advocating transparency, specificity of how this might apply within a health context remains ambiguous due to a number of reasons including: conflicting policy priorities, lack of policy prescription and alignment at local levels and limited professional and institutional capacity in local government.^32^ Additionally, this study also emphasised a holistic approach and need for direct engagement with planning professionals to provide opportunities for effective use of the planning system to promote healthier environments.

### Strengths and limitations

While studies have explored the use of the planning system to regulate HFTs in England^33^ and have reported on the decisions made by the Planning Inspectorate,^26^ this article is the first to bring together the views of a range of professionals about the appeals process. The strength of this work is the first in-depth examination of the planning appeals process in relation to HFTs in England capturing the varying priorities, understandings and perspectives of the range of actors involved.

A key limitation of this research was the limited recruitment due to the COVID-19 pandemic response and lockdowns (2020-21). We had originally planned to interview at least 32 participants with stakeholders from LAs across England, as well as businesses and Planning Inspectors. Recruitment proved much slower and more challenging than anticipated. The COVID-19 pandemic and its impact on working conditions and resources meant that many LAs were unable to engage with the project due to their increased workloads, time constraints and other added pressures. We, therefore, changed our initial data collection method to incorporate a questionnaire/survey to maximise responses from LAs across England. Although this provided responses and captured a little more information in relation to the appeal process, only seven additional members of LA staff completed the survey. This method also had its own limitations, being less in depth and ‘rich’ compared to interviews yet did provide LA staff with an anonymous platform to share information and gave added perspective that would otherwise not have been achieved. On discussing this with our stakeholders, it became clear that the continuing effects of the COVID-19 pandemic would have made it difficult to engage with LAs in any given capacity, particularly those working in public health and planning departments. Additionally, businesses were either closed or under extreme pressures at the time of the data collection and we were unable to recruit any businesses to the study.

### Implications for practice

Through the findings of this study, we summarised six suggestions for successfully defending a refusal of planning permission at appeal, outlined in Table 2.

**Table 2:** Suggestions for successfully defending a refusal of planning permission at appeal

***(1) Local Authorities should ensure that a clean and robust local plan is in place and applied correctly*** As stated, reference to a local plan is often necessary for a successful appeal, however, if there is any ambiguity to its application, defending a refusal becomes substantially more difficult. Likewise, if Local Planning Policy and Supplementary Planning Documents are not applied correctly, a defence becomes more difficult, even if the evidence behind a line of argument is robust.
***(2) Having adequate time and staff capacity to deal with appeals cases/acknowledging time required to deal with an appeal.*** Those new to the appeals process were often surprised by the amount of time and effort required to defend a refusal. Without sufficient capacity a defence is very likely to fall short.
***(3) Having access to accurate, robust, and up to date local information to use in the appeals case.*** If there is a debate surrounding the accuracy of any information cited a defence can be undermined.
***(4) Having firm commitment from elected members and senior management from various professional groups (such as Planning and Public Health).*** A defence is more likely to succeed if professionals have permission from senior management to make it a priority, rather than relying on their enthusiasm to work beyond what would typically be expected of them.
***(5) Good lines of communication with relevant local groups & communities interested in the appeal.*** Although subjective evidence such as the opinions of the local residents are perceived as anecdotal, communication with the relevant groups can lead to invaluable information, and their passion for a cause can help drive a defence forward.
***(6) Using clear, concise language that is accessible across professional groups.*** Public Health and Planning professions have their own jargon which is often lost in translation when these groups communicate, the use of clear and accessible language can help prevent these problems and thus accelerate the appeal process.

## Conclusion

Successfully defending a planning decision by a LA requires a range of issues to be in place; from having the appropriate planning policies to the correct application of these planning processes. It requires commitment from staff, building on communication between professional groups and clear lines of communication. Training on the importance of health in planning was identified; the Office for Health Improvement and Disparities (previously PHE) have commissioned and are developing this work. Further work is underway by this research group to develop and evaluate practical guides for use by both Planning and Public Health professionals working in this area.
